# Inequalities in the use of insecticide-treated nets by pregnant women in Ghana, 2011 and 2017

**DOI:** 10.1186/s12936-022-04388-z

**Published:** 2022-12-09

**Authors:** Eugene Budu, Joshua Okyere, Felix Mensah, Simon Agongo Azure, Abdul-Aziz Seidu, Edward Kwabena Ameyaw, Bright Opoku Ahinkorah

**Affiliations:** 1 Korle Bu Teaching Hospital, P. O. Box, 77, Accra, Ghana; 2 Department of Population and Health, University of Cape Coast, Cape Coast, Ghana; 3 Department of Nursing, College of Health Sciences, Kwame Nkrumah University of Science and Technology, Kumasi, Ghana; 4 Department of Data Science and Economic Policy, University of Cape Coast, Cape Coast, Ghana; 5 Population and Reproductive Health Division, School of Public Health, University of Port Harcourt, Choba, Port Harcourt, Nigeria; 6 Department of Community Health, College of Health, Yamfo, Ghana; 7 Centre for Gender and Advocacy, Takoradi Technical University, Takoradi, Ghana; 8 College of Public Health, Medical and Veterinary Sciences, James Cook University, Townsville, Australia; 9 Institute of Policy Studies and School of Graduate Studies, Lingnan University, Hong Kong, China; 10 L & E Research Consult Ltd., Wa, Upper West Region Ghana; 11 School of Public Health, Faculty of Health, University of Technology Sydney, Sydney, Australia

**Keywords:** Insecticide-treated net, Pregnant women, Ghana, Multiple indicator cluster survey

## Abstract

**Background:**

Pregnant women and children are the most vulnerable group of people usually affected by malaria. The use of insecticide-treated nets is one of the proven interventions for mitigating malaria and its associated deaths in endemic regions, including Ghana. Meanwhile, there is limited evidence on the extent of inequality in insecticide-treated nets use by pregnant women in Ghana. This study assessed the inequalities in insecticide-treated nets use by pregnant women in Ghana.

**Methods:**

Data from the 2011 and 2017 versions of the Ghana Multiple Indicator Cluster Surveys were used. The 2019 updated World Health Organization (WHO) HEAT software (version 3.1) was used for all analyses. Four equity stratifiers were employed to disaggregate insecticide-treated nets use by pregnant women in Ghana. These are economic status, level of education, place of residence, and sub-national region. Four measures were used to compute inequality namely Difference (D), Population Attributable risk (PAR), Population Attributable Fraction (PAF) and Ratio (R).

**Results:**

The analyses indicated a rise in pregnant women’s insecticide-treated nets use from 32.6% in 2011 to 49.7% in 2017. Except sub-national region, all the factors showed mild inequality in insecticide-treated nets use. For instance, with respect to the economic status of pregnant women, only a slight inequality was exhibited by one of the simple measures in both 2011 (R = 0.3; 95% UI = 0.2–0.6) and 2017 (R = 0.5; 95% UI = 0.3–0.7). Marginal inequality in insecticide-treated nets use was noted in 2011 (R = 0.6; 95% UI = 0.5–0.9) and 2017 (R = 0.8; 95% UI = 0.6–0.9) for level of education. In the same vein, slight inequality was realized with respect to place of residence in 2011 (R = 0.4; 95% UI = 0.3–0.6) and 2017 (R = 0.6; 95% UI = 0.5–0.7). For sub-national region, both simple (D = 50.5; 95% UI = 30.7–70.4) and complex (PAF = 91.3; 95% UI = 72.3–110.3) measures demonstrated substantial inequality in 2011. In the case of 2017, considerable inequality in insecticide-treated nets use occurred (D = 58; 95% UI = 42.2–73.8, PAF = 51.9; 95% UI = 36.2–67.6).

**Conclusion:**

In conclusion, insecticide-treated nets utilization by pregnant Ghanaian women increased between 2011 and 2017. The findings show that Ghana’s Ministry of Health in collaboration with anti-malarial non-governmental organizations must review patterns of insecticide-treated nets distribution and intensify advocacy among educated pregnant women, those in urban settings and the rich, to assuage the magnitude of inequality.

## Background

Malaria remains a life-threatening disease and is caused by parasites that enter persons through the bites of infected *Anopheles* mosquitoes [1]. An estimated 241 million cases of malaria were recorded worldwide in 2020 [1]. Although everyone is susceptible to malaria, pregnant women and children are the most disadvantaged [2]. According to the World Health Organization (WHO) [3], maternal malaria interferes with the growth of the fetus, thus increasing the risk of premature delivery and low birth weight. The use of insecticide-treated nets (ITNs) is one of the interventions that has shown to reduce malaria infection and contribute to reduction of malaria induced deaths in the endemic regions [4]. A systematic review on ITNs use revealed that, ITNs reduce the prevalence of *Plasmodium falciparum* malaria by 17% [5]. In Ghana, household access to ITNs alone contributed to 7.1 percentage point reduction in the self-reported malaria among women [6].

A couple of factors account for inequalities in the use of ITNs. A study in Ghana by Bawuah and Ampaw [7] revealed that place of residence and wealth predict ownership and use of ITN. Similarly, Luukwa et al. [8] argued that children and secondary educated pregnant women from poor rural households in one region in Ghana had the highest usage of ITNs, relative to pregnant women with other characteristics. Also, in Malawi, the odds of utilizing ITN was low among women with no education, those with primary education and the poor [9].

Despite efforts to promote ownership and utilization of ITNs, there is still a mismatch between ITN ownership and use. For instance, a study by Kanmiki et al. [10], revealed that 79% of respondents owed ITNs, while 62% of the ITN owners used them. Assessing the inequalities and disparity of ITNs use in Ghana will be relevant to policy-makers in redesigning strategies to increase usage and aid greatly towards the reduction of malaria burden in the society, particularly among pregnant women. The aim of this study, therefore, is to assess the inequalities in the use of by pregnant women in Ghana from 2011 to 2017 using the Multiple Indicator Cluster Survey (MICS) data.

## Methods

### Study design and data source

Data from the 2011 and 2017 versions of the Ghana MICS were used. MICS is a nationally representative cross-sectional survey that aims to monitor the situation of women and children by capturing their information on health, education, social protection, environment, domestic violence along with the socio-economic, demographic and geographic characteristics at the individual and household levels. The sampling frame of the MICS is based on the population census preceding the survey. As such, the sampling frames for the 2011 and 2017 MICS were from the 2010 Ghana Population and Housing Census. MICS employs a multistage, stratified cluster probability sampling design to establish a representative sample of households at the national and regional level. Within each region, the urban and rural areas were defined as the main sampling strata. Household samples were selected in the following stages: (i) within each stratum, a specified number of census enumeration areas (EAs) or clusters were selected systematically with probability proportional to size (then listing of the household was done for the selected EAs) and (ii) the sample of households were selected from the sampled EAs. The complete sampling procedure has been elaborated in the final reports of the 2011 and 2017 MICS of Ghana.

### Variables of interest

The outcome variable for the study was whether pregnant women sleep under ITNs or not. Pregnant women who slept under ITNs were categorized as “yes”, whilst those who did not were classified otherwise “no”. Four stratifiers were used to assess inequality in sleeping under ITNs: economic status measured by wealth quintile (quintile 1, 2, 3, 4, 5), level of education (no education, primary education, secondary/higher education), place of residence (rural, urban), and sub-national region (Western, Central, Greater Accra, Volta, Eastern, Ashanti, Brong Ahafo, Northern, Upper West, Upper East). Wealth quintile was derived by employing Principal Component Analysis (PCA). Education was measured by highest level of formal education completed [11, 12].

### Statistical analyses

The 2019 updated WHO HEAT software (version 3.1) was used for all analyses [13]. Four equity stratifiers were employed to disaggregate ITNs use among pregnant women in Ghana. These are economic status, level of education, residence, and sub-national region. Estimates and uncertainty intervals (UIs) of sleeping under ITNs with respect to the aforementioned stratifiers were computed. Four measures were used to compute inequality namely Difference (D), Population Attributable risk (PAR), Population Attributable Fraction (PAF) and Ratio (R). Two of these are simple unweighted measures (D, R) and two are complex weighted measures (PAR, PAF). At the same time, R and PAF are relative measures whereas D and PAR are absolute measures. Summary measures were considered because the WHO [14] has indicated that both absolute and relative summary measures are essential for generating policy driven findings [13]. Unlike simple measures, the complex ones take size of categories inherent in a sub-population into account. The WHO has extensively elaborated the procedure for generating summary measures [13, 15, 16].

In calculating D in economic status, poorest pregnant women who slept under ITNs were deducted from richest pregnant women who slept under ITNs. For level of education, D was computed as pregnant women who sleep under ITNs and had “no formal education” minus pregnant women who slept under ITNs and had “secondary/higher education”, while D in place of residence was about inequality between rural and urban residents. With respect to sub-national region, D was computed by deducting the region with lowest estimate from the region with the highest estimate. The R for the variables was computed with ordered responses such as level of education and economic status as the difference between the most-disadvantaged sub-group (lowest quintile and uneducated) and the most-advantaged sub-group (highest quintile and secondary/higher  education). PAR was derived by computing the difference in estimate of pregnant women who slept under ITN in relation to the reference category (*yref*) and overall average of the prevalence of women who slept under ITN. With respect to the ordered variables, *y*_*ref*_ referred to the most-advantaged subgroups. In the case of sub-national region, which was non-ordered, y_ref_ meant the region with the lowest estimate. The PAF was gauged by distributing PAR by the overall average μ, and multiplied by 100 (PAF = [PAR/μ] * 100). Zero (0) PAF or PAR means no inequality, whilst a higher value indicates a relatively higher inequality. Variation in women who slept under ITN was explored by making reference to the 95% UIs of the survey years. Absence of overlap in the UIs means that a statistically significant difference existed between the UIs and vice versa.

### Ethical approval

Ethics approval for this study was not required since the data is secondary and is available in the public domain. More details regarding the data and ethical standards are available at: https://mics.unicef.org/surveys.

## Results

### Trends in pregnant women sleeping under insecticide-treated nets by different inequality dimensions, 2011 and 2017

The analyses indicated a rise in pregnant women’s ITN use from 32.6% in 2011 to 49.7% in 2017 (see Table 1). In both surveys, ITNs use among pregnant women was well pronounced among poorest women manifesting in 51.4% and 65.1% in 2011 and 2017, respectively. Pregnant women who had no formal education dominated in ITNs use in 2011 (42.2%) and 2017 (56.8%). It was also observed that most rural pregnant women used ITNs in both surveys; 43.6% in 2011 and 61.5% in 2017 as compared with the urban pregnant women (18.8% and 35.5% in 2011 and 2017, respectively). Across the regions, it was evident that in 2011, ITNs use was profound in the Eastern region (62.3%), but was least reported among pregnant women in the Greater Accra region (11.8%). In the 2017 survey, use of ITNs dominated in the Volta region (69.4%), but was least pronounced in the Greater Accra region, where only 17.5% prevalence was recorded.

**Table 1:** Trends in pregnant women's insecticide-treated nets use by different inequality dimensions, 2011 and 2017

Dimension	2011 (32.6%)		2017 (49.7%)	
	Sample	% (95% CI)	Sample	% (95% CI)
Economic status				
Quintile 1 (poorest)	185	51.4[44.3, 58.4]	187	65.1[54.1, 74.6]
Quintile 2	150	49.7[39.4, 60.1]	196	59.1[46.1, 71]
Quintile 3	150	31.9[22.9, 42.5]	180	50.8[37.4, 64.2]
Quintile 4	166	12.9[7.8, 20.5]	188	43.2[33.2, 53.9]
Quintile 5 (richest)	160	15.8[8.7, 26.9]	182	29.4[20.5, 40]
Education				
No formal education	236	42.2[34.5, 50.2]	235	56.8[46.4, 66.6]
Primary school	166	33[24.4, 43]	165	60.2[48.8, 70.5]
Secondary/higher education	409	26.9[21.2, 33.4]	534	43.4[37.2, 49.7]
Place of residence				
Rural	451	43.6[38.6, 48.7]	510	61.5[54.3, 68.3]
Urban	360	18.8[13.2, 26.1]	423	35.5[29.2, 42.2]
Sub-national region				
Ashanti	186	21.3[12.3, 34.4]	263	47[36.4, 57.8]
Brong Ahafo	72	36.1[23.6, 50.9]	92	60.9[46.3, 73.8]
Central	81	29.3[21.2, 39.1]	69	44.5[31, 58.8]
Eastern	71	62.3[44.2, 77.6]	91	53.6[39.3, 67.3]
Greater Accra	107	11.8[5, 25.5]	92	17.5[8.8, 31.7]
Northern	86	37.9[30.7, 45.7]	113	49.1[36.5, 61.9]
Upper East	32	50.5[42.3, 58.6]	29	75.5[62.8, 84.9]
Upper West	22	41.2[31.4, 51.8]	22	61.8[48.6, 73.4]
Volta	74	57.8[46.4, 68.4]	76	69.4[48.4, 84.5]
West	80	21.5[11.5, 36.5]	88	52.5[36.1, 68.3]

### Inequality indices of estimates of the use of  insecticide-treated nets by pregnant women, 2011 and 2017

Table 2 illustrates the indices of estimates of factors associated with insecticide-treated net utilization among pregnant women. The indices comprised simple (D and R) and complex (PAF and PAR) measures. Except region of residence, all the factors showed mild inequality in insecticide-treated net use. For instance, with respect to economic status of the pregnant women, only a slight inequality was exhibited by one of the simple measures in both 2011 (R = 0.3) and 2017 (R = 0.5). Marginal inequality in insecticide-treated net use was noted in 2011 (R = 0.6) and 2017 (R = 0.8). In the same vein, slight inequality was realized with respect to region of residence in 2011 (R = 0.4) and 2017 (R = 0.6). With respect to region, both simple (D = 50.5) and complex (PAF = 91.3) measures demonstrated substantial inequality in 2011. In the case of 2017, considerable inequality in insecticide-treated net occurred (D = 58, PAF = 51.9).

**Table 2:** Inequality indices of estimates of factors associated with pregnant women sleeping under insecticide-treated nets, 2011 and 2017

Dimension	2011			2017		
	Estimate	Lower Bound	Upper Bound	Estimate	Lower Bound	Upper Bound
Economic status						
Difference (D)	− 35.6	− 47.1	− 24.2	− 35.7	− 50	− 21.5
Population Attributable Fraction (PAF)	0	− 19.6	19.6	0	− 12.7	12.7
Population Attributable Risk (PAR)	0	− 6.4	6.4	0	− 6.3	6.3
Ratio (R)	0.3	0.2	0.6	0.5	0.3	0.7
Level of education						
Difference (D)	− 15.3	− 25.3	− 5.3	− 13.4	− 25.4	-1.4
Population Attributable Fraction (PAF)	0	− 16	16	0	− 11.1	11.1
Population Attributable Risk (PAR)	0	− 5.2	5.2	0	− 5.5	5.5
Ratio (R)	0.6	0.5	0.9	0.8	0.6	0.9
Place of residence						
Difference (D)	− 24.8	− 33	− 16.6	− 26.1	− 35.6	− 16.5
Population Attributable Fraction (PAF)	0	− 8.9	8.9	0	− 6.2	6.2
Population Attributable Risk (PAR)	0	− 2.9	2.9	0	− 3.1	3.1
Ratio (R)	0.4	0.3	0.6	0.6	0.5	0.7
Sub-national region						
Difference (D)	50.5	30.7	70.4	58	42.2	73.8
Population Attributable Fraction (PAF)	91.3	72.3	110.3	51.9	36.2	67.6
Population Attributable Risk (PAR)	29.7	23.6	35.9	25.8	18	33.6
Ratio (R)	5.3	2.2	12.7	4.3	2.2	8.4

## Discussion

Several studies have established the critical role of ITNs in malaria prevention, particularly in sub-Saharan Africa and Ghana [17, 18]. Yet, there are existing inequalities in terms of ITNs utilization, thereby putting some sub-populations at a disadvantage. The study examined the inequalities in ITNs by pregnant women in Ghana. The findings revealed that ITNs by pregnant women has increased from 32.6% in 2011 to 49.7% in 2017. This figures are greater than the prevalence recorded in sub-Saharan Africa (30.6%) and Madagascar (10.1%) [17]. The results, however, corroborate an earlier study conducted in Ghana [19] which reported a prevalence of 49.2%. Probably, the increase in the utilization of ITNs between 2011 and 2017 could be attributed to the implementation of the National Malaria Control Programme (NMCP), which provides free mass and continuous distribution of ITNs in hospitals and schools [20, 21]. Although the results suggest an increase in the utilization of ITNs in Ghana between the period under study, the current prevalence of ITNs utilization (49.7%) raises concerns as to whether Ghana would be able to achieve the WHO target of 90% decline in the incidence of malaria infections and mortality due to malaria by the year 2030 [22]. Hence, there is the need for policy and intervention reviews in order to identify and forge new strategies to improve ITNs utilization in Ghana.

Across both survey, the utilization of ITNs was higher among women from poorer households as compared to women from richer households. This finding is somewhat surprising because previous studies have revealed that women from higher wealth indexed households are more likely to own ITNs and consequently, being more likely to utilize it [23–25]. Nevertheless, the result is analogous to the findings of a related study conducted in Ghana which found that although women from higher wealth indexed households were more likely to own ITNs, the utilization was rather higher among women from poorer households [10]. A plausible explanation for this finding could be that, unlike women from richer households, women from poorer wealth households are unable to afford alternative preventive methods such as intermittent preventive treatment, repellents and aerosol sprays [10, 26]. The inequality estimate revealed that there was marginal inequality in the utilization of ITNs between 2011 and 2017. Probably, this could be as a result of the pro-poor nature of the NMCP which mostly target poorer households with free ITNs distribution.

Contrary to findings from previous studies that found higher ITNs utilization among women in urban areas [27, 28], the study found that rural dwelling pregnant women had higher ITNs utilization in both 2011 and 2017 as compared to their counterparts in rural areas. The result is, however, consistent with the findings from studies conducted in Ghana [29, 30] and Myanmar [31]. The reasons for this finding is unclear, however, it could be that rural dwelling women struggle to afford malaria treatment and, therefore, prioritize preventive measures such as ITNs use, far more than their counterparts residing in urban areas [32]. With respect to inequality analysis, the study revealed that there was marginal inequality in the utilization of ITNs. Thus, suggesting that Ghana has been able to narrow the rural–urban inequalities in terms of ITNs utilization over time.

Concerning the education dimension, it was revealed that ITNs utilization was higher among women who had no formal education compared to those who had formal education across both survey points. Similar findings have been reported by a related study from Ghana [30]. A plausible explanation for this could be that, women with no formal education often perceive themselves to be highly vulnerable to malaria infection, possibly receiving encouragement to utilize ITNs as compared to those with formal education. Dadzie et al. [30] also argue that women with formal or higher education have greater access to information about alternative malaria prevention methods. Consequently, they tend to practice better environmental hygiene and/or adopt alternatives to ITNs, hence, explaining the lower ITNs utilization among women with no formal education, and the corresponding higher ITNs utilization among those with formal education. However, there was marginal educational-based inequality with regards to the utilization of ITNs over time.

Regarding the region of residence, the findings indicate that women from Greater Accra region were the least to utilize ITNs in both the 2011 and 2017 survey points. However, in 2011, the utilization of ITNs was higher among women in the Eastern region; whilst women in the Volta region dominated in terms of ITNs use in 2017. There was substantial inequality in utilization of ITNs in both 2011 and 2017. The reasons for the substantial regional inequalities in ITNs utilization and the differences over time are unclear. However, it is possible that the low ITNs utilization in the Greater Accra region may be due to the characteristics of the region. Women in the Greater Accra region tend to be urbanites and also have higher formal education; all of these factors have been found to be associated with lower utilization of ITNs [27–30]. Another possible explanation could be that, Ghana’s ITNs programme has generally be pro-poor and pro-rural in nature, hence, explaining the low utilization of ITNs in the Greater Accra region.

### Policy implications

From a policy perspective, there is the need for Ghana to review the free, mass distribution of ITNs component of the NMCP. The study reveals that the current pro-poor and pro-rural nature of ITNs utilization may be triggering inequalities in the utilization of ITNs. Therefore, a second look and revision in the current NMCP could improve ITNs utilization in Ghana. The findings also underscore the need to enhance health educational messages about the importance of using ITNs in urban areas and in the Greater Accra region.

### Strengths and limitations

The study used nationally representative data, hence, making the findings generalizable to the entirety of Ghana. Also, the use of the HEAT software was appropriate for estimating various inequality estimates for the various dimensions. This adds to the rigor and validity of the study methodology. Nonetheless, there are some limitations that should be considered when interpreting the study findings. As a study that relied on secondary data, it was not possible for us to account for other important factors such as cultural norms that could play key role in women’s utilization of ITNs. Also, given that information on ITNs was self-reported, there is the possibility of recall and self-reporting bias.

## Conclusion

ITNs utilization has increased from 32.6% in 2011 to 49.7% in 2017. Women with no formal education, those who resided in rural residences, and women from poorer households have higher utilization of ITNs. There is marginal inequality in the utilization of ITNs across the dimensions of place of residence, wealth index and educational attainment. However, there is substantial regional inequalities in the utilization of ITNs. The findings show that Ghana’s Ministry of Health in collaboration with anti-malarial non-governmental organization might have to review patterns of insecticide-treated net distribution and intensify advocacy among the educated pregnant women, those in urban settings and the rich, to assuage the magnitude of inequality.

## Data Availability

The data is freely available at https://mics.unicef.org/surveys once permission is sought and granted by UNICEF.

## Abbreviations

HEAT: Health equity assessment toolkit
ITN: Insecticide-treated net
MICS: Multiple indicator cluster survey
NMCP: National malaria control programm
PAR: Population attributable risk
PAF: Population attributable fraction
UIs: Uncertainty intervals
WHO: World Health Organization
